# Retraction: Androgen receptor promotes gastric cancer cell migration and invasion via AKT-phosphorylation dependent upregulation of matrix metalloproteinase 9

**DOI:** 10.18632/oncotarget.28736

**Published:** 2025-05-29

**Authors:** Bao-gui Zhang, Tao Du, Ming-de Zang, Qing Chang, Zhi-yuan Fan, Jian-fang Li, Bei-qin Yu, Li-ping Su, Chen Li, Chao Yan, Qin-long Gu, Zheng-gang Zhu, Min Yan, Bingya Liu

**Affiliations:** ^1^Shanghai Key Laboratory of Gastric Neoplasms, Department of Surgery, Shanghai Institute of Digestive Surgery, Ruijin Hospital, Shanghai Jiao Tong University School of Medicine, Shanghai, China; ^2^Department of Surgery, Shanghai East Hospital, Tongji University School of Medicine, No 150 Jimo Road, Shanghai, China; ^*^These authors contributed equally to this work

**This article has been retracted:** Oncotarget has completed its investigation of this article. Oncotarget’s Image Forensics analysis identified multiple instances of image manipulations. These include duplications and overlaps of transwell assay images between Figures 2B and 5C, 5D. Further duplications and overlaps were identified between Figures 1A (IHC of human tumors) and 6C (mouse tumors), along with internal overlaps within Figures 1A and 2B. Additionally, multiple manipulations, appearing as repeated sections within the same image, were found in Figures 2C, 5A, and 6C. Given these findings, the editorial decision has been made to retract the paper. The first author, Dr. Zhang, acknowledged the errors, however, despite our attempts to confirm the retraction with the authors, no response was received.

Original article: Oncotarget. 2014; 5:10584–10595. 10584-10595. https://doi.org/10.18632/oncotarget.2513

